# Quality-by-Design-Driven Nanostructured Lipid Scaffold of Apixaban: Optimization, Characterization, and Pharmacokinetic Evaluation

**DOI:** 10.3390/pharmaceutics16070910

**Published:** 2024-07-08

**Authors:** Kiran Patil, Nayan Gujarathi, Charu Sharma, Shreesh Ojha, Sameer Goyal, Yogeeta Agrawal

**Affiliations:** 1 Shri Vile Parle Kelavani Mandal’s Institute of Pharmacy, Dhule 424001, Maharashtra, India; kiranpharma143@gmail.com (K.P.); nayan.gujarathi@svkm.ac.in (N.G.); sameer.goyal@svkm.ac.in (S.G.); 2 Department of Internal Medicine, College of Medicine and Health Sciences, United Arab Emirates University, Al-Ain P.O. Box 15551, United Arab Emirates; charusharma@uaeu.ac.ae; 3 Department of Pharmacology and Therapeutics, College of Medicine and Health Sciences, United Arab Emirates University, Al-Ain P.O. Box 15551, United Arab Emirates; shreeshojha@uaeu.ac.ae

**Keywords:** Apixaban, nanostructured lipid carriers (NLCs), 2^3^ full factorial design, high-pressure homogenization, anticoagulant

## Abstract

Apixaban, an anticoagulant, is limited in its efficacy due to poor solubility, low bioavailability, and extensive metabolism. This study investigates the application of nanostructured lipid carriers (NLCs) to enhance the bioavailability of Apixaban. NLCs were prepared using the high-pressure homogenization method. The influence of independent variables, viz., the amount of Tween 80, HPH pressure, and the number of HPH cycles, were studied using a 2^3^ factorial design. The average particle size, PDI, zeta potential, and entrapment efficiency of the optimized NLCs were found to be 232 ± 23 nm, with 0.514 ± 0.13 PDI and zeta potential of about −21.9 ± 2.1 mV, respectively. Additionally, concerning the thermal and crystallographic properties of the drug, the NLCs showed drug entrapment without altering its potency. The in-vitro drug release studies revealed an immediate release pattern, followed by sustained release for up to 48 h. In-vivo pharmacokinetic experiments demonstrated that Apixaban-loaded NLCs exhibited higher values of t_1/2_ (27.76 ± 1.18 h), AUC_0–∞_ (19,568.7 ± 1067.6 ng·h/mL), and C_max_ (585.3 ± 87.6 ng/mL) compared to free drugs, indicating improved bioavailability. Moreover, a decrease in the elimination rate constant (Kel) reflected the sustained effect of Apixaban with NLCs. NLCs offer improved oral absorption rates and enhanced therapeutic impact compared to free drugs, potentially reducing dose frequency and improving patient outcomes.

## 1. Introduction

In recent years, direct oral anticoagulants (DOACs) such as Apixaban and Rivaroxaban have gained significant attention for their effectiveness in preventing and treating thromboembolic disorders. Both Apixaban and Rivaroxaban have been successfully used in clinical practice due to their predictable pharmacokinetics, lower risk of bleeding compared to traditional anticoagulants, and no requirement for routine monitoring [1,2]. Apixaban is a potent and specific factor Xa (FXa) inhibitor. Factor Xa is a crucial clotting factor, categorized in the S1 peptidase family, responsible for venous thrombi and arterial sclerosis by converting prothrombin into thrombin. The formation of thrombin leads to deposits of fibrin and the activation of platelets, indirectly contributing to the formation of clots. Apixaban inhibits the action of FXa by reducing thrombin production, thereby indirectly suppressing the platelet aggregation induced by thrombin and preventing clot formation [3,4]. This mechanism makes Apixaban a promising alternative to conventional anticoagulant therapies such as vitamin K antagonists, aspirin, and low-molecular-weight heparin, due to its enhanced benefit-to-risk ratio.

Despite its therapeutic potential, Apixaban faces significant challenges related to its poor water solubility, low oral bioavailability, and substantial first-pass metabolism. Over 50% of the orally administered dose is excreted unchanged from the body, and about 25% undergoes hepatic metabolism without producing active metabolites [5]. Additionally, the conventional dosage forms of Apixaban can cause adverse effects due to non-specific therapeutic action and low drug concentration at the target site.

To address these limitations, novel targeted drug delivery systems such as nanostructured lipid carriers (NLCs) have emerged as promising alternatives. NLCs offer several advantages over traditional formulations. Their biocompatible lipid matrix allows for sustained drug release, while their small size enables them to bypass the hepatic first-pass metabolism, facilitating an effective drug accumulation at target sites. This enhances bioavailability and therapeutic outcomes [6,7,8]. Moreover, NLCs can improve the solubility and stability of poorly water-soluble drugs by optimizing their pharmacokinetic and pharmacodynamics profiles.

Recent research has highlighted the potential of NLCs in enhancing drug delivery. For instance, studies have demonstrated that NLCs can improve the oral bioavailability of various drugs, including poorly soluble compounds, by enhancing their solubility and stability [9]. Additionally, NLCs have been shown to provide sustained drug release, reducing dosing frequency and improving patient compliance [10,11,12]. These findings emphasize the importance of NLCs as a versatile carrier system for drug delivery.

The present study employs the Quality by Design (QbD) approach to prepare and optimize Apixaban NLCs. The QbD methodology is a systematic approach to pharmaceutical development that emphasizes understanding the process and controlling variability to ensure a consistent product quality [13,14]. This approach involves identifying critical quality attributes (CQAs) and critical process parameters (CPPs) and using the design of experiments (DoE) to optimize the formulation and manufacturing process. By applying QbD principles, this study aims to develop a robust and reproducible Apixaban NLC formulation with improved therapeutic efficacy.

The formulation was characterized for compatibility, surface morphology, entrapment efficiency, X-ray diffraction (PXRD), and drug release. Thermal analysis and stability studies were performed to estimate the stability of Apixaban-loaded NLCs. Moreover, an in vivo pharmacokinetic study in Wistar rats was conducted to assess the pharmacokinetic parameters of the optimized formulation, to confirm the enhanced bioavailability and therapeutic potential of the NLC formulation.

The overall objective of this research is to explore the novel application of NLCs to overcome the limitations of Apixaban, a potent anticoagulant with significant therapeutic potential. The use of the Quality by Design (QbD) approach ensures the development of a robust and optimized NLC formulation, with improved bioavailability and therapeutic outcomes. Moreover, this novel approach provides a controlled and sustained release profile, potentially reducing dosing frequency and improving patient compliance. The biocompatibility and safety of the GRAS lipids used minimize the risk of adverse reactions, making the formulation suitable for long-term use. Additionally, the method is simple, scalable, and cost-effective, utilizing readily available lipids and standard equipment. The findings of this study contribute to the scientific evidence supporting the use of NLCs as a promising drug delivery system for poorly soluble drugs, offering significant advantages over traditional formulations.

## 2. Material and Methods

Apixaban was received as a gift sample from Pinnacle Life Science, Mumbai, India. Glycerol Monostearate, Oleic Acid, and Tween 80 were purchased from Loba Chemicals Pvt Ltd., Mumbai, India. All the reagents and chemicals used are of analytical grade. Design-Expert^®^ V11 (Stat-Ease, Minneapolis, MN, USA) software was used to optimize the formulation.

### 2.1. Screening of Lipids

The most important step in an NLC formulation is to perform a lipid screening for the solubility study. This aims to identify the most suitable lipids for the encapsulation of the active ingredient into the NLCs. To screen for suitable lipids, 10 mg incremental amounts of the drug were added separately to both liquid and solid molten lipids and stirred continuously until supersaturation was reached. The maximum amount of the drug that each lipid could dissolve was then determined. This was achieved by measuring the solubility spectrophotometrically at 280 nm [15].

### 2.2. Formulation of Apixaban NLCs

The Apixaban NLCs were prepared using a hot high-pressure homogenization technique. A total 1.5% lipid was used, with a solid-to-liquid lipid ratio of 70:30. The solid lipid was melted, into which 10 mg of Apixaban was accurately weighed and incorporated using a magnetic stirrer with a hot plate (Q-20A, REMI, Mumbai, India). Subsequently, the liquid lipid was added, and the mixture was heated to 80 °C, resulting in the formation of a transparent lipid phase. To this, an aqueous surfactant solution of Tween 80 was then slowly added at the same temperature with constant stirring, until a milky white pre-emulsion was formed. The pre-emulsion was then exposed to 9 to 11 cycles of high-pressure homogenization (T 25 Ultra-Turrax, IKA^®^, Osaka, Japan), at 15,000 rpm for 15 min, with the pressure ranging from 600 to 800 bar, leading to the formation of NLCs [16,17]. 

### 2.3. Optimization Using a Full Factorial Design 

The Quality by Design (QbD) approach was used to optimize the NLCs using Design Expert^®^ software (Stat-Ease, Minneapolis, MN, USA). A 2^3^ full factorial design was used to interpret the impact of independent variables on the response variable. The surfactant concentration (*X*1), HPH pressure (*X*2), and HPH cycle (*X*3) were changed between −1 and +1 levels to optimize the independent variables (Table 1) against the response variables, particle size (*Y*1), and %EE (*Y*2) [18,19]. The polynomial equation and 2D and 3D surface response plots were used to interpret the simultaneous effect of the independent variables on the response variables.

(1) $$y=b_{0}+b_{1}X1+b_{2}X2+b_{3}X3+b_{12}X1X2+b_{13}X1X3+b_{23}X2X3$$

### 2.4. Particle Size, Polydispersity Index, and Zeta Potential 

The nanocarrier size was estimated using the dynamic light scattering technique (DLS) with a Zetasizer (Nano ZS90, Malvern, Cambridge, UK). The NLCs dispersion was diluted with distilled water before analysis. The DLS provided data on both the polydispersity index (PDI) and the mean diameter of the NLCs. A zeta potential measurement was performed to estimate the surface charge of the NLCs, using the Helmholtz–Smoluchowski equation. These measurements were conducted at 25 °C, with a 23 V/m electric field [17,20,21,22].

### 2.5. Entrapment Efficiency (%)

The investigation focused on determining the amount of Apixaban entrapped within the NLCs. This aimed to quantify the exact drug amount encapsulated within the NLCs relative to the total drug quantity used in the formulation. Briefly, a 2 mL sample of the NLCs dispersion was centrifuged at 4 °C and 10,000 rpm for 30 min. Subsequently, the drug content was estimated spectrophotometrically at 278 nm. The percent entrapment efficiency (%EE) was then calculated using Equation (2) [23,24,25].

(2) $$\%\mathrm{EE}=\frac{Wt.\;of\;drug\;used\;in\;formulation\;-\;Wt.\;of\;unbound\;drug\;in\;supernatant}{Wt.\;of\;drug\;used\;in\;formulation}\times 100$$

### 2.6. Lyophilization of NLCs

The Apixaban NLCs aqueous dispersion with 2% mannitol as a cryoprotectant was frozen for 24 h at –75 °C in a refrigerator for pre-freezing. The samples were freeze-dried by employing a lab freeze-dryer (SP VirTis BenchTop Pro; SP Scientific, Ipswich, UK). The lyophilization was continued for a further 70–72 h. Then, the vials were sealed with rubber closures for further investigation [26,27].

### 2.7. Thermal Analysis by DSC

A solid-state characterization of pure Apixaban and lyophilized Apixaban NLCs was analyzed by DSC. The phase transition temperature and energy were verified by DSC studies (Mettler-Toledo, Columbus, OH, USA; STAR DSC 1). An exact 2 mg sample was weighed precisely and placed in aluminium pans before being sealed. The scanning was carried out between 30 °C to 250 °C, with a heating rate of 10 °C/min and nitrogen flow rate of 50 mL/min [28,29].

### 2.8. Transmission Electron Microscopy 

Transmission electron microscopy (TEM) (FEI, Hillsboro, OR, USA; Tecnai G2 F20) was used to examine the nanostructure of Apixaban NLCs. A carbon-coated grid was placed on top of a diluted NLC drop that had been applied on a paraffin sheet for one minute. The NLCs adhered to the carbon substrate. The filter paper was used to remove the remaining NLC drop. Afterward, the grid was left for 10 s on a drop of phosphotungstic acid (1%). The residual solution was absorbed using filter paper, and before the TEM analysis, the sample was air-dried [30,31].

### 2.9. In Vitro Drug Release 

The timely release of an entrapped drug is a critical step in the formulation development of a novel dosage form. Various factors such as the structure and composition of the nanolipoidal carrier have a significant impact on the pattern of drug release. Therefore, it was investigated exactly how Apixaban was released from the NLCs in vitro. Optimized Apixaban NLCs were tested for drug release in phosphate buffer solution (PBS, pH 6.8) utilizing the dialysis bag method [32]. The dialysis bag method was chosen for its simplicity and effectiveness in providing a controlled environment for studying the release kinetics of the drug from the developed NLCs. This method allows for the continuous monitoring of drug release and helps in understanding release mechanisms, which are critical for predicting the in vivo behavior of the drug-loaded NLCs. The dialysis bags (molecular weight cut-off: 12–14 kDa) were prepared by soaking them overnight in distilled water to remove any preservatives and ensure proper hydration. Each bag was then filled with 2 mL of the NLC dispersion and sealed at both ends. The sealed dialysis bags were immersed in phosphate-buffered saline at 37 ± 0.5 °C and subjected to magnetic agitation at 50 rpm. At predefined intervals up to 72 h, 0.5 mL of the solution was withdrawn and filtered using a 0.22 µm filter (Millipore, Burlington, MA, USA). A fresh buffer solution was added to maintain sink conditions. The filtrates were diluted and analyzed using a UV spectrophotometer at 278 nm [33,34,35].

### 2.10. Pharmacokinetic Studies

The in vivo pharmacokinetics of Apixaban was studied for both a conventional suspension and NLCs loaded with the drug to investigate the in vivo fate of Apixaban. The protocol and experimental procedure as outlined were approved by the IAEC and CPCSEA (Protocol No. RCPIPER/IAEC/203) committees of the R. C. Patel Institute of Pharmaceutical Education and Research, Shirpur.

#### 2.10.1. Experimental Design

Sprague Dawley rats weighing about 200–250 g were selected to conduct the experiments. Acrylic cages were used to house the animals and provided with pelleted chow meal and potable water. The environment in relation to temperature and humidity remained steady at 25 °C and 50% RH, with a cycle of 12 h for darkness and light. The animals fasted for 12 h before treatment and 4 h afterward, with only access to drinking water. There were two groups, i.e., a test and control, each with six animals. An equivalent dose of the drug (5 mg/kg), based on the rat’s body weight, was administered to the test group. However, a suspension of Apixaban dispersed in a solution of sodium CMC (0.5%) was administered to the control group [36,37].

#### 2.10.2. Pharmacokinetic Data Analysis

Blood samples at each of the allocated time marks (0.5, 1, 2, 4, 8, 12, 24, 48, and 72 h) were obtained in an aliquot by each animal’s retro-orbital plexus, and subsequently, the animals were euthanized. The samples were analyzed by the RP-HPLC method previously developed by Subramanian et al. [38]. The mobile phase A was comprised of buffer phase and acetonitrile 90:10 *v*/*v*, and water and acetonitrile (10:90 *v*/*v*) were used for for mobile phase B. Chromatographic separation was performed on a C18 column (250 × 4.6 mm, 5 μm), with a flow rate of 1.0 mL/min, detection at 280 nm, an injection volume of 50 μL, and a column temperature of 40 °C. The non-compartmental pharmacokinetic model was used to determine the plasma concentration–time plot for the Apixaban NLCs and drug molecules. The parameters were assessed using Kinetica 5.0 Software (Thermo Fisher Scientific, Waltham, MA, USA). The concentration–time plot of the drug was used to compute the primary pharmacokinetic characteristics, in particular, C_max_, T_max_, and AUC_0–∞_ along with the t_1/2_ and mean residence time [39]. 

### 2.11. Accelerated Stability Studies 

The stability and shelf life of the NLCs must be ensured before commercialization in the pharmaceutical market. The minimum requirements include a shelf life of one year, sufficient drug-holding ability, and maintaining the size of the nanocarriers throughout storage. In conformity to this, the leakage of drug, nanocarrier size, and zeta potential had to be characterized. The size of the nanocarriers, polydispersity index, and %EE were used to assess the NLCs’ stability over three months. The influence of temperature on the characteristics of the NLCs was also investigated. The stability studies were executed at two different temperature conditions: at 25 °C ± 2 °C/60% RH ± 5% RH (accelerated stability conditions), and 5 °C ± 3 °C (refrigerated conditions) [40].

### 2.12. Statistical Analysis

The results were presented as mean ± SEM. The statistical significance was assessed using one-way ANOVA followed by Dunnett’s test, with a 95% confidence interval. A *p*-value of less than 0.05 was considered statistically significant. The statistical analysis was conducted using GraphPad Prism Software (Version 7).

## 3. Result and Discussion

### 3.1. Screening of Lipids

The solubility screening of the drug in solid and liquid lipids revealed a higher relative distribution of Apixaban in GMS, reaching 39.2 ± 0.15 mg, compared to other solid lipids. Conversely, Oleic acid exhibited superior solubility, with 52.6 ± 0.32 mg of the drug compared to other liquid lipids. The screening of both solid and liquid lipids for optimal drug solubility led to the selection of GMS and Oleic acid as the most promising for use in Apixaban NLCs. Additionally, both lipids are biocompatible, biodegradable, and affordable GRAS excipients.

### 3.2. Optimization of Apixaban NLCs by Factorial Design

A two-level (2^3^) full factorial design was applied to optimize Apixaban NLCs, considering Tween 80 concentration (*X*1), HPH pressure (*X*2), and HPH cycles number (*X*3) as factors, and size of nanocarrier (*Y*1) and efficiency of drug entrapment (*Y*2) as a response (Table 2). Polynomial models were employed to estimate the statistical significance and simultaneous interaction of the variables. The quadratic polynomial model effectively predicted and elucidated the interaction between the critical quality attributes (CQAs) and the corresponding responses. This finding was further supported by the F-test (*p* < 0.05), indicating the suitability of the model. The results of the full factorial design are summarized in Table 3. The high correlation coefficient (R^2^) values suggest the strong fit of the quadratic polynomial model. Additionally, an insignificant lack of fit (*p* > 0.05) indicates pure error, further confirming the model’s appropriateness. Moreover, the small difference (less than 0.2) between predicted R^2^ and adjusted R^2^ values compared to actual R^2^ values underscores the accuracy of the chosen model. 

#### 3.2.1. Impact of Independent Variables on Size of Nanocarrier (*Y*1)

The following is the polynomial equation for the size of the nanocarriers:

Particle Size = +300.13 + 55.88*A* + 2.87*B* + 17.37*C* − 1.38*AB* + 4.6*BC* + 6.38*ABC* (3)

**Figure 1:**
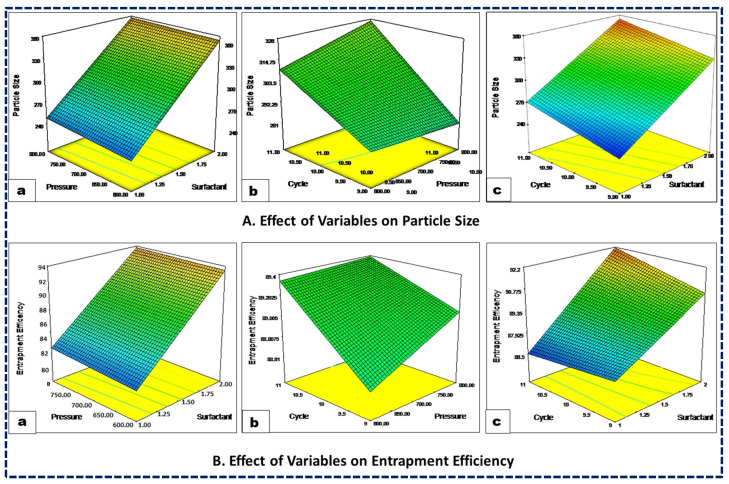
Three-dimensional response surface plots showing the influence of surfactant concentration (**a**), HPH pressure (**b**), and HPH cycles (**c**) on the CQAs, viz., the particle size (**A**) and entrapment efficiency (**B**) of the NLCs.

The particle size of the nanoformulations significantly influences formulation success by enhancing absorption, encapsulation, and reducing systemic clearance, thereby increasing drug exposure. Additionally, the physicochemical properties of the drug are closely related to the formulation particle size. The particle-size distribution across all the experimental trials consistently ranged from 220 nm to 385 nm (Table 3), with a polynomial equation expressed as Equation (1). The coded equation and 3D response curve (Figure 1A) illustrate that an increased surfactant concentration leads to a decreased particle size, likely due to a reduced interfacial tension between the lipid and aqueous phases. Similarly, higher homogenization pressure results in a smaller particle size due to increased compression, turbulence, and cavitation during the homogenization process. Moreover, an increase in the number of homogenization cycles tends to reduce particle size, as the NLCs undergo repeated cycles of homogenization. Furthermore, the color gradients visually demonstrate the impact of the independent variables on the response variables, making it easier to interpret how changes in surfactant concentration, pressure, and cycle affect particle size and entrapment efficiency. In the case of particle size, the color gradient from blue to red indicates the change in particle size, with blue representing smaller particle sizes and red representing larger particle sizes. Similarly, for %EE the color gradient from blue to red indicates the change in entrapment efficiency, with blue representing lower efficiency and red representing higher efficiency (Figure 1).

#### 3.2.2. Impact of Independent Variables on the Efficiency of Drug Entrapment (*Y*2) 

The following is the polynomial equation for entrapment efficiency:

(4) $$EE\left(\%\right)=+89.10+2.26A+0.12B+0.27C+0.54AC-0.097BC+0.58ABC$$

The effectiveness of a drug delivery system depends on its ability to achieve an optimal concentration of drug at the target site. Their ability to encapsulate large quantities of drugs within colloidal carriers significantly enhances their effectiveness. In the present study, the %EE ranged between 86.0% and 92.7%. The impact of variations in the independent variables on the %EE is illustrated in Figure 1B. An increased surfactant concentration markedly enhanced the %EE of the drug by promoting an accumulation of Tween 80 interfacial film on the globular surface and thereby increasing its encapsulation within the lipid matrix. Conversely, homogenization pressure exhibited an inverse relationship with the %EE, especially beyond its critical threshold. High pressure led to a decreased %EE, possibly due to increased energy causing NLCs to rupture and subsequent drug expulsion. Similarly, an increase in HPH cycles also tended to reduce the %EE, as the drug leaked out of the carriers with each cycle. Similar results were also reported by other studies of NLCs encapsulating drugs [41,42,43]. 

### 3.3. Size of Nanocarriers and Zeta Potential of Optimized Apixaban-Loaded NLCs

**Figure 2:**
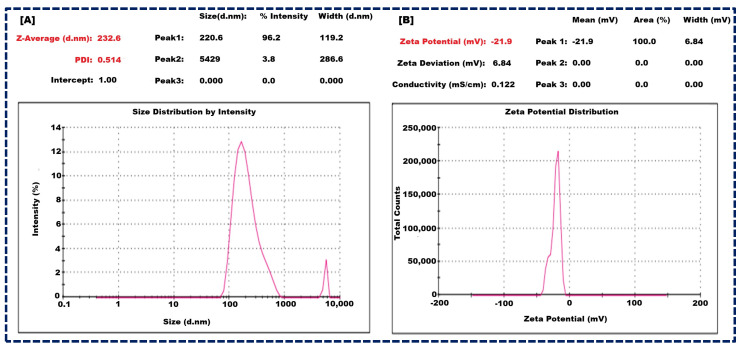
Size of nanocarrier of NLCs (**A**), and Zeta potential of NLCs (**B**).

The optimized batch of NLCs exhibited promising characteristics and showed a mean particle size of 232 ± 23 nm (Figure 2A), indicating their nano-scale nature. Furthermore, the polydispersity index (PDI) was measured at 0.514 ± 0.13, suggesting a relatively narrow size distribution, which is crucial for uniform drug delivery. Moreover, the zeta potential was recorded at −21.9 ± 2.1 mV (Figure 2B), which falls within the optimal range of −30 mV to +30 mV for colloidal stability. The zeta potential is due to the combined effects of GMS, Oleic acid, and Tween 80. GMS provides structural integrity and amphiphilic characteristics, Oleic acid contributes negatively charged carboxylate ions, and Tween 80 offers steric stabilization. These components work synergistically to stabilize the NLCs and prevent aggregation, ensuring a stable and effective drug delivery system. These findings are pivotal for enhancing drug encapsulation efficiency, sustained release, and overall therapeutic efficacy [21,44].

### 3.4. Differential Scanning Calorimetry 

**Figure 3:**
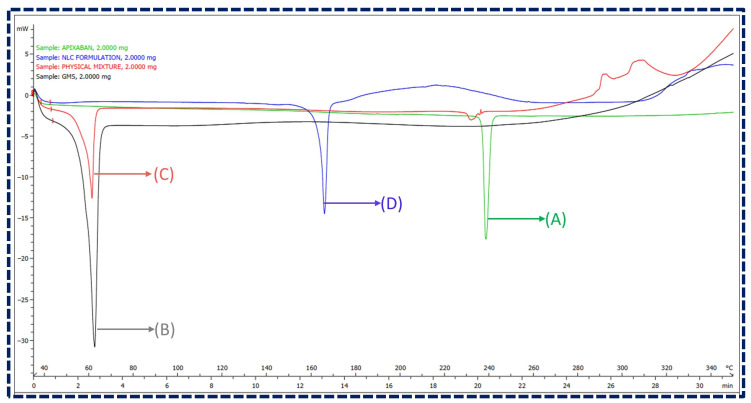
Overlay of DSC thermograms for (A) Apixaban, (B) GMS, (C) physical mixture of Apixaban and GMS, and (D) Apixaban-loaded NLCs.

A DSC analysis was employed to ensure the absence of interactions between the drug and excipients. The overlay plot of DSC thermograms of Apixaban, GMS, their physical mixture (1:1 ratio), and Apixaban NLCs are illustrated in Figure 3. The overlay depicted Apixaban and GMS as exhibiting distinct endothermic peaks at 238.58 °C and 61.57 °C, respectively. The physical mixture has shown no observable shift in the position of these peaks, indicating no chemical interactions between the components. Moreover, the thermogram of lyophilized NLCs revealed two endothermic peaks, one at around 61.49 °C, attributed to the glass transition temperature of the lipidic phase, and another at 163.44 °C, corresponding to mannitol, a cryoprotectant used in the lyophilization process. However, the absence of an endothermic peak corresponding to Apixaban in the NLCs thermogram suggests its molecular inclusion within the lipid matrix, indicating an amorphous state. This signifies the successful encapsulation of Apixaban in the NLCs, confirming their potential for stable drug delivery without chemical interactions [45,46].

### 3.5. Transmission Electron Microscopy

**Figure 4:**
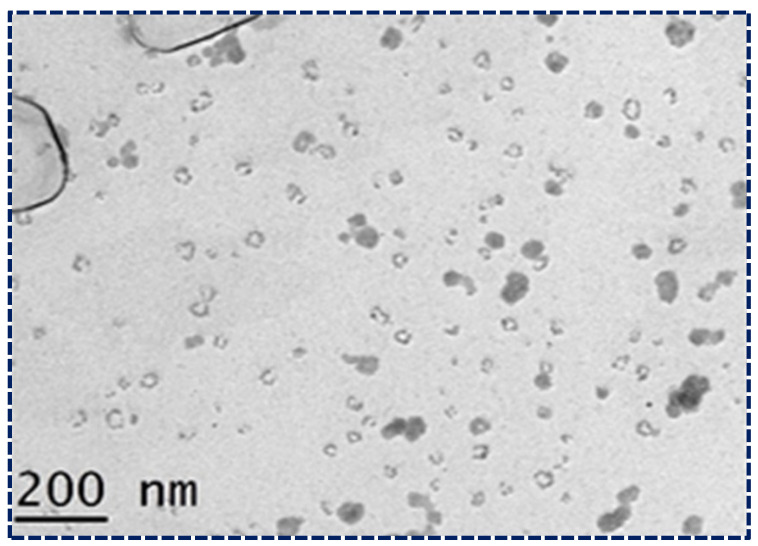
Morphological study of NLCs loaded with Apixaban under TEM.

The surface morphology of the optimized NLCs was studied using transmission electron microscopy (TEM) (Figure 4). The TEM image provided critical insights into the structural characteristics of the NLCs. The image showed that the NLCs had a spherical morphology with a uniform size distribution and no agglomeration, indicating that the formulation process was successful in producing stable NLCs. Additionally, the size of the NLCs was consistently below 200 nm, which is crucial for enhancing bioavailability and ensuring efficient drug delivery. These characteristics, observed in the TEM images, confirm that the NLCs possess the desired properties for potential therapeutic applications. The spherical shape and uniform size distribution contribute to the stability and predictability of the NLCs’ behavior in biological systems, further validating their suitability for the present study. This detailed examination of the TEM images underscores the importance of morphological analysis in the development and optimization of NLC formulations [47].

### 3.6. Lyophilization of Apixaban NLCs 

The Apixaban NLCs were lyophilized to improve their storage stability. A benchtop freeze drier was used to lyophilize the lipid dispersion. The lyophilization was performed for an optimized batch at −75 °C, with 2% mannitol as a cryoprotectant. Cryoprotectants play an important role by means of a reduction in the aggregation of nanocarriers during lyophilization. Dry, porous, and friable properties were discovered in the lyophilized powder after 72 h. At 76 mTorr, the vacuum was maintained [48,49].

### 3.7. Drug Release Studies from NLCs 

**Figure 5:**
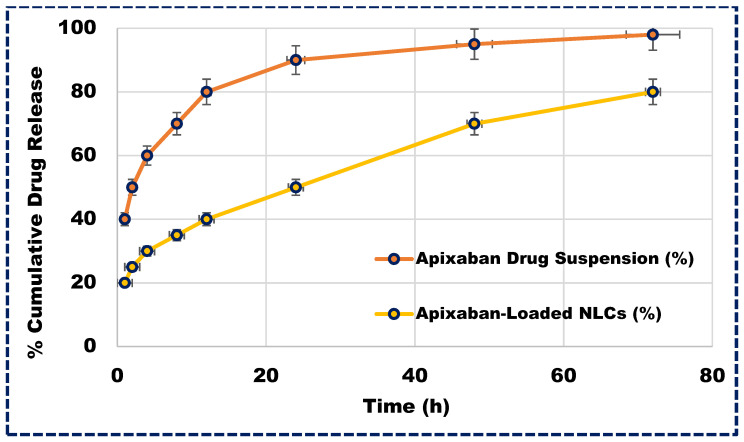
The in vitro release profile of Apixaban-loaded NLCs and Apixaban suspension.

Understanding the release profile of drugs is not only essential for developing innovative formulations but also integral to the standard quality control tests required for regulatory approval. Apixaban-loaded NLCs exhibited a biphasic release pattern, as depicted in Figure 5. Initially, a burst release occurred within the first two hours, followed by a sustained release of the drug. This release pattern involved sequential events, starting with the diffusion of unentrapped medication from the aqueous surfactant phase (26.33%), followed by drug diffusion from the NLC surface and subsequently from the lipid matrix. This gradual release may be attributed to the gradual penetration of the aqueous diffusion medium into the lipid layer, leading to the dissolution and slow diffusion of Apixaban. Approximately 96.33% of the Apixaban was released from the NLCs over 72 h, indicating the sustained release potential of the delivery system and thereby enhancing its therapeutic efficacy. 

**Figure 6:**
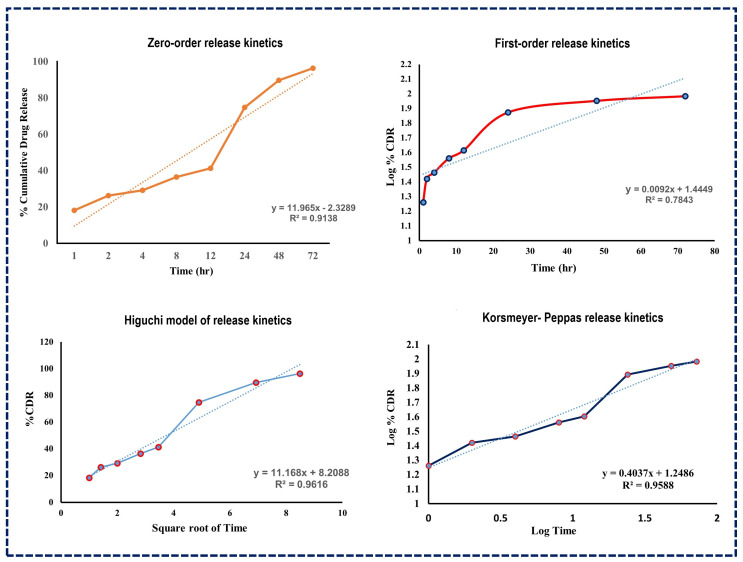
Drug release kinetics models for Apixaban-loaded NLCs.

The subsequent analysis using various release kinetics models, as illustrated in Figure 6, revealed R-squared values for several models including the Higuchi model (R^2^ = 0.9616), Korsmeyer–Peppas model (R^2^ = 0.9588), first-order kinetic model (R^2^ = 0.7843), and zero-order kinetic model (R^2^ = 0.9138). The high R-squared value of the Higuchi model suggests that drug release from NLCs follows a diffusion process, indicating a split into immediate and prolonged release phases [50].

### 3.8. Pharmacokinetic Study

**Figure 7:**
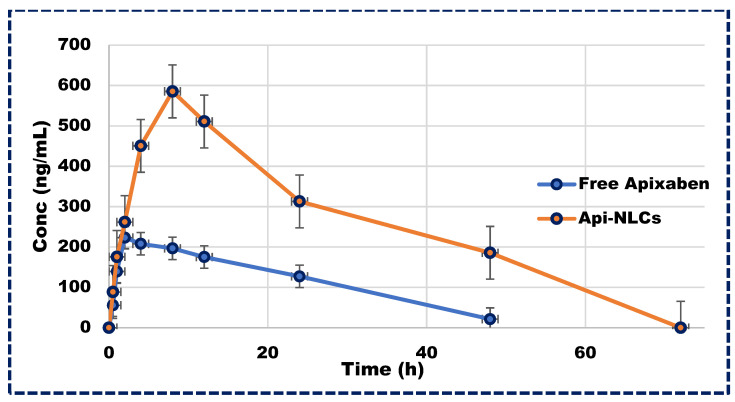
Plasma concentration vs. time profile in Wistar rats for Apixaban NLCs and free drug.

Following a single dose of Apixaban NLCs, plasma concentrations of the drug remained above therapeutic levels for 72 h, in contrast to the rapid excretion of the free drug within 24 h, as illustrated in Figure 7. The pharmacokinetics parameters of Apixaban NLCs are represented in Table 4, exhibiting significantly different C_max_ and T_max_ values compared to the free drug in plasma profiles. Moreover, the total elimination rate constant (K_ele_) for the free drug exceeded that of the NLCs, leading to a greater half-life (t_1/2_) for Apixaban NLCs. Additionally, the area under the curve (AUC_0–∞_) was markedly higher for Apixaban NLCs (*p* < 0.001) compared to its free form. This significant improvement in the pharmacokinetics of Apixaban NLCs may be attributed to the nano-sized particle’s ability to bypass the lymphatic system, thereby circumventing first-pass metabolism and prolonging drug exposure, emphasizing the therapeutic potential of NLC-based delivery systems [51,52].

### 3.9. Accelerated Stability Studies 

Particle size measurement, polydispersity index, and drug entrapment efficiency in NLCs were utilized to assess the stability of the formulated optimized NLCs over a period of 3 months, focusing on the impact of storage temperature. Stability testing was conducted as per Q1A (R^2^) ICH guidelines at refrigerated conditions (5 °C ± 3 °C) and accelerated conditions (25 °C ± 2 °C/60% RH ± 5% RH), with comparisons made to the initial analysis performed immediately after the preparation of the optimized batch of NLCs. The concise characterization data under different temperature conditions is presented in Table 5. The results of the stability testing indicated that the improved batch remained considerably stable under refrigerated conditions [53,54].

## 4. Conclusions

Apixaban’s poor bioavailability is attributed to high protein binding and rapid metabolism. The present research successfully formulated Apixaban NLCs to augment solubility, bioavailability, and sustained drug release. The ObD approach-based optimized NLCs exhibited promising physiochemical characteristics. The NLCs exhibited a spherical shape and nanometric size, with high drug content. In vitro drug release studies confirmed a sustained drug release over 72 h. A thermal and spectral analysis validated drug–excipient compatibility. Accelerated stability testing further affirmed the formulation stability. In vivo pharmacokinetic studies demonstrated the enhanced bioavailability of Apixaban NLCs, evidenced by an increased drug concentration in the systemic circulation (AUC_0–∞_). This suggests the potential of NLCs for dose reduction and improved therapeutic effectiveness due to enhanced carrier efficiency, nano-sized dimensions, improved solubilization, and reduced metabolism. In conclusion, formulating Apixaban NLCs emerges as a promising strategy for enhancing bioavailability and therapeutic efficacy.

## Figures and Tables

**Table 1:** Factors and response variables with levels and constraints using Design Expert^®^.

Independent Variables	Levels	
	Low (−1)	High (+1)
*X*1: Tween 80 concentration (%)	1	2
*X*2: HPH pressure (bar)	600	800
*X*3: HPH cycles (number)	9	11
Response variables	Constraint	
*Y*1: Size of nanocarriers) nm)	Minimum	
*Y*2: Entrapment efficiency (%)	Maximum	

**Table 2:** Two-level (2^3^) full factorial design matrix and responses of Abixaban-loaded NLCs.

Batch	*X*1	*X*2	*X*3	*Y*1	*Y*2
	Tween 80 Conc. (%)	HPH Pressure (bar)	HPH Cycles (Number)	Particle Size (nm)	Entrapment Efficiency (%)
F1	2	800	9	330	90.21
F2	1	800	11	265	86.00
F3	2	600	9	349	90.90
F4	2	600	11	360	91.54
F5	1	800	9	232	91.90
F6	1	600	9	220	86.32
F7	1	600	11	260	87.14
F8	2	800	11	385	92.78

**Table 3:** Statistical summary report given by DoE software.

Response	F-Value	*p*-Value	R^2^	Adjusted R^2^	Predicted R^2^	Model Significance *
Particle Size	761.05	0.0277	0.9998	0.9985	0.9860	Significant
Entrapment Efficiency	62.08	0.0371	0.9971	0.9900	0.9923	Significant

* *p* value of 0.05 or less is considered significant.

**Table 4:** Pharmacokinetic characteristics of Apixaban and Apixaban-loaded NLCs.

Parameters	Free Form	Apixaban-Loaded NLCs
C_max_ (ng/mL)	232.2 ± 49	585.3 ± 87.6
T_max_ (h)	2.5 ± 0.18	8 ± 0.3
t_1/2_ (h)	11.59 ± 0.22	27.76 ± 1.18 ***
AUC_0–∞_ (ng·h)/mL	5463.97 ± 321.6	19,568.7 ± 1067.6 ***

*** *p* < 0.001 for Apixaban-loaded NLCs in comparison to free form of drug.

**Table 5:** Accelerated stability studies of Apixaban-loaded NLCs.

Sampling Time (Days)	Size of Nanocarriers (nm)	Polydispersity Index	Entrapment Efficiency %
At time of preparation	232.6	0.514	91.9
Storage at room temperature (28–30 °C)			
15	240.2	0.556	88.33
30	281.5	0.603	89.74
Storage in refrigerator (5 °C ± 3 °C)			
15	235.9	0.542	90.1
30	250.8	0.586	89.89

## Data Availability

The original contributions presented in the study are included in the article.

## References

[1] John A., Gorzelanny C., Bauer A.T., Schneider S.W., Bolenz C. (2018). Role of the Coagulation System in Genitourinary Cancers: Review. Clin. Genitourin. Cancer.

[2] Wei S., Sawhney A., Khandait H., Meda A., Gupta V., Jain R. (2023). An Update on Applications and Limitations of Direct Oral Anticoagulants. Egypt. J. Intern. Med..

[3] Greig S.L., Garnock-Jones K.P. (2016). Apixaban: A Review in Venous Thromboembolism. Drugs.

[4] Koehl J.L., Hayes B.D., Al-Samkari H., Rosovsky R. (2020). A Comprehensive Evaluation of Apixaban in the Treatment of Venous Thromboembolism. Expert Rev. Hematol..

[5] Byon W., Garonzik S., Boyd R.A., Frost C.E. (2019). Apixaban: A Clinical Pharmacokinetic and Pharmacodynamic Review. Clin. Pharmacokinet..

[6] Ali J., Bhavna, Ali M., Baboota S. (2008). Patents on Nanoparticulate Drug Delivery Systems—A Review. Recent Pat. Drug Deliv. Formul..

[7] Jain K.K. (2020). Role of Nanobiotechnology in Drug Delivery. Methods Mol. Biol..

[8] Friedrich R.P., Pöttler M., Cicha I., Lyer S., Janko C., Alexiou C. (2016). Novel Nanoparticulate Drug Delivery Systems. Nanomedicine.

[9] El-Shenawy A.A., Mahmoud R.A., Mahmoud E.A., Mohamed M.S. (2021). Intranasal In Situ Gel of Apixaban-Loaded Nanoethosomes: Preparation, Optimization, and In Vivo Evaluation. AAPS PharmSciTech.

[10] Elmowafy M., Al-Sanea M.M. (2021). Nanostructured Lipid Carriers (NLCs) as Drug Delivery Platform: Advances in Formulation and Delivery Strategies. Saudi Pharm. J..

[11] Mahor A.K., Singh P.P., Gupta R., Bhardwaj P., Rathore P., Kishore A., Goyal R., Sharma N., Verma J., Rosenholm J.M. (2023). Nanostructured Lipid Carriers for Improved Delivery of Therapeutics via the Oral Route. J. Nanotechnol..

[12] Mehta M., Bui T.A., Yang X., Aksoy Y., Goldys E.M., Deng W. (2023). Lipid-Based Nanoparticles for Drug/Gene Delivery: An Overview of the Production Techniques and Difficulties Encountered in Their Industrial Development. ACS Mater. Au.

[13] Al-Mohaya M., Mesut B. (2024). Quality by Design Approach for Novel Drug Delivery Systems. Introduction to Quality by Design (QbD).

[14] Mohseni-Motlagh S.F., Dolatabadi R., Baniassadi M., Baghani M. (2023). Application of the Quality by Design Concept (QbD) in the Development of Hydrogel-Based Drug Delivery Systems. Polymers.

[15] Kovačević A.B., Müller R.H., Keck C.M. (2020). Formulation Development of Lipid Nanoparticles: Improved Lipid Screening and Development of Tacrolimus Loaded Nanostructured Lipid Carriers (NLC). Int. J. Pharm..

[16] Souto E.B., Müller R.H. (2006). Investigation of the Factors Influencing the Incorporation of Clotrimazole in SLN and NLC Prepared by Hot High-Pressure Homogenization. J. Microencapsul..

[17] Lakhani P., Patil A., Wu K.-W., Sweeney C., Tripathi S., Avula B., Taskar P., Khan S., Majumdar S. (2019). Optimization, Stabilization, and Characterization of Amphotericin B Loaded Nanostructured Lipid Carriers for Ocular Drug Delivery. Int. J. Pharm..

[18] Agarwal S., HariKumar S.L., Negi P., Upadhyay N., Garg R. (2021). Quetiapine Fumarate Loaded Nanostructured Lipid Carrier for Enhancing Oral Bioavailability: Design, Development and Pharmacokinetic Assessment. Curr. Drug Deliv..

[19] Agrawal Y., Petkar K.C., Sawant K.K. (2010). Development, Evaluation and Clinical Studies of Acitretin Loaded Nanostructured Lipid Carriers for Topical Treatment of Psoriasis. Int. J. Pharm..

[20] Bahari L.A.S., Hamishehkar H. (2016). The Impact of Variables on Particle Size of Solid Lipid Nanoparticles and Nanostructured Lipid Carriers; A Comparative Literature Review. Adv. Pharm. Bull..

[21] Zhang H.-W., Dang Q., Zhang Z.-W., Wu F.-S. (2017). Development, Characterization and Evaluation of Doxorubicin Nanostructured Lipid Carriers for Prostate Cancer. J. BUON.

[22] Sun M., Zhu Z., Wang H., Han C., Liu D., Tian L., Yang X., Pan W. (2017). Surface Density of Polyarginine Influence the Size, Zeta Potential, Cellular Uptake and Tissue Distribution of the Nanostructured Lipid Carrier. Drug Deliv..

[23] Masjedi M., Azadi A., Heidari R., Mohammadi-Samani S. (2020). Nose-to-Brain Delivery of Sumatriptan-Loaded Nanostructured Lipid Carriers: Preparation, Optimization, Characterization and Pharmacokinetic Evaluation. J. Pharm. Pharmacol..

[24] Hanna P.A., Ghorab M.M., Gad S. (2019). Development of Betamethasone Dipropionate-Loaded Nanostructured Lipid Carriers for Topical and Transdermal Delivery. Antiinflamm. Antiallergy Agents Med. Chem..

[25] Madan J.R., Khobaragade S., Dua K., Awasthi R. (2020). Formulation, Optimization, and in Vitro Evaluation of Nanostructured Lipid Carriers for Topical Delivery of Apremilast. Dermatol. Ther..

[26] Managuli R.S., Reddy M.S., Koteshwara K.B., Mutalik S. (2021). Enteric Coating of Nanostructured Lipid Carriers (NLCs) and Enteric Coating of Hard Gelatin Capsules Filled with NLCs: Feasibility Studies. Pak. J. Pharm. Sci..

[27] Nimtrakul P., Sermsappasuk P., Tiyaboonchai W. (2020). Strategies to Enhance Oral Delivery of Amphotericin B: A Comparison of Uncoated and Enteric-Coated Nanostructured Lipid Carriers. Drug Deliv..

[28] Rodrigues da Silva G.H., Ribeiro L.N.M., Mitsutake H., Guilherme V.A., Castro S.R., Poppi R.J., Breitkreitz M.C., de Paula E. (2017). Optimised NLC: A Nanotechnological Approach to Improve the Anaesthetic Effect of Bupivacaine. Int. J. Pharm..

[29] Wang L., Luo Q., Lin T., Li R., Zhu T., Zhou K., Ji Z., Song J., Jia B., Zhang C. (2015). PEGylated Nanostructured Lipid Carriers (PEG–NLC) as a Novel Drug Delivery System for Biochanin A. Drug Dev. Ind. Pharm..

[30] Wang J., Wang H., Xia Q. (2018). Ubidecarenone-Loaded Nanostructured Lipid Carrier (UB-NLC): Percutaneous Penetration and Protective Effects Against Hydrogen Peroxide-Induced Oxidative Stress on HaCaT Cells. Int. J. Mol. Sci..

[31] Zhuang C.-Y., Li N., Wang M., Zhang X.-N., Pan W.-S., Peng J.-J., Pan Y.-S., Tang X. (2010). Preparation and Characterization of Vinpocetine Loaded Nanostructured Lipid Carriers (NLC) for Improved Oral Bioavailability. Int. J. Pharm..

[32] Wolska E., Szymańska M. (2023). Comparison of the In Vitro Drug Release Methods for the Selection of Test Conditions to Characterize Solid Lipid Microparticles. Pharmaceutics.

[33] Rebouças-Silva J., Tadini M.C., Devequi-Nunes D., Mansur A.L., Silveira-Mattos P.S., Oliveira C.I.D., Formiga F.R., Berretta A.A., Marquele-Oliveira F., Borges V.M. (2020). Evaluation of in Vitro and in Vivo Efficacy of a Novel Amphotericin B-Loaded Nanostructured Lipid Carrier in the Treatment of Leishmania Braziliensis Infection. Int. J. Nanomed..

[34] Dumont C., Bourgeois S., Fessi H., Dugas P.-Y., Jannin V. (2019). In-Vitro Evaluation of Solid Lipid Nanoparticles: Ability to Encapsulate, Release and Ensure Effective Protection of Peptides in the Gastrointestinal Tract. Int. J. Pharm..

[35] Teeranachaideekul V., Souto E.B., Junyaprasert V.B., Müller R.H. (2007). Cetyl Palmitate-Based NLC for Topical Delivery of Coenzyme Q10—Development, Physicochemical Characterization and in Vitro Release Studies. Eur. J. Pharm. Biopharm..

[36] Liu S.J., Liu J.W., Cong J.Z., Tong L., Zhang Y., Li X.M., Hou J. (2018). Preparation of Neogambogic Acid Nanoliposomes and Its Pharmacokinetics in Rats. J. Coll. Physicians Surg. Pakistan.

[37] Shete H., Chatterjee S., De A., Patravale V. (2013). Long Chain Lipid Based Tamoxifen NLC. Part II: Pharmacokinetic, Biodistribution and in Vitro Anticancer Efficacy Studies. Int. J. Pharm..

[38] Subramanian V.B., Katari N.K., Dongala T., Jonnalagadda S.B. (2020). Stability-indicating RP-HPLC Method Development and Validation for Determination of Nine Impurities in Apixaban Tablet Dosage Forms. Robustness Study by Quality by Design Approach. Biomed. Chromatogr..

[39] Tirumalesh C., Suram D., Dudhipala N., Banala N. (2020). Enhanced Pharmacokinetic Activity of Zotepine via Nanostructured Lipid Carrier System in Wistar Rats for Oral Application. Pharm. Nanotechnol..

[40] Patil K.D., Bagade S.B., Bonde S.C. (2020). In-Vitro and Ex-Vivo Characterization of Novel Mannosylated Gelatin Nanoparticles of Linezolid by Quality-by-Design Approach. J. Drug Deliv. Sci. Technol..

[41] Cunha S., Costa C.P., Loureiro J.A., Alves J., Peixoto A.F., Forbes B., Sousa Lobo J.M., Silva A.C. (2020). Double Optimization of Rivastigmine-Loaded Nanostructured Lipid Carriers (NLC) for Nose-to-Brain Delivery Using the Quality by Design (QbD) Approach: Formulation Variables and Instrumental Parameters. Pharmaceutics.

[42] Shimojo A.A.M., Fernandes A.R.V., Ferreira N.R.E., Sanchez-Lopez E., Santana M.H.A., Souto E.B. (2019). Evaluation of the Influence of Process Parameters on the Properties of Resveratrol-Loaded NLC Using 22 Full Factorial Design. Antioxidants.

[43] Rathod V.R., Shah D.A., Dave R.H. (2020). Systematic Implementation of Quality-by-Design (QbD) to Develop NSAID-Loaded Nanostructured Lipid Carriers for Ocular Application: Preformulation Screening Studies and Statistical Hybrid-Design for Optimization of Variables. Drug Dev. Ind. Pharm..

[44] Mu H., Holm R. (2018). Solid Lipid Nanocarriers in Drug Delivery: Characterization and Design. Expert Opin. Drug Deliv..

[45] Sarhadi S., Gholizadeh M., Moghadasian T., Golmohammadzadeh S. (2020). Moisturizing Effects of Solid Lipid Nanoparticles (SLN) and Nanostructured Lipid Carriers (NLC) Using Deionized and Magnetized Water by in Vivo and in Vitro Methods. Iran. J. Basic Med. Sci..

[46] Chen X., Zhang Y., Zhao P., Chen Y., Zhou Y., Wang S., Yin L. (2020). Preparation and Evaluation of PEGylated Asiatic Acid Nanostructured Lipid Carriers on Anti-Fibrosis Effects. Drug Dev. Ind. Pharm..

[47] Proetto M.T., Rush A.M., Chien M.-P., Abellan Baeza P., Patterson J.P., Thompson M.P., Olson N.H., Moore C.E., Rheingold A.L., Andolina C. (2014). Dynamics of Soft Nanomaterials Captured by Transmission Electron Microscopy in Liquid Water. J. Am. Chem. Soc..

[48] Agrawal Y.O., Mahajan U.B., Mahajan H.S., Ojha S. (2020). Methotrexate-Loaded Nanostructured Lipid Carrier Gel Alleviates Imiquimod-Induced Psoriasis by Moderating Inflammation: Formulation, Optimization, Characterization, In-Vitro and In-Vivo Studies. Int. J. Nanomed..

[49] Liu K., Sun J., Wang Y., He Y., Gao K., He Z. (2008). Preparation and Characterization of 10-Hydroxycamptothecin Loaded Nanostructured Lipid Carriers. Drug Dev. Ind. Pharm..

[50] Ritger P.L., Peppas N.A. (1987). A Simple Equation for Description of Solute Release II. Fickian and Anomalous Release from Swellable Devices. J. Control. Release.

[51] Haider M., Abdin S.M., Kamal L., Orive G. (2020). Nanostructured Lipid Carriers for Delivery of Chemotherapeutics: A Review. Pharmaceutics.

[52] Raj S.B., Chandrasekhar K.B., Reddy K.B. (2019). Formulation, In-Vitro and In-Vivo Pharmacokinetic Evaluation of Simvastatin Nanostructured Lipid Carrier Loaded Transdermal Drug Delivery System. Future J. Pharm. Sci..

[53] Kaur N., Sharma K., Bedi N. (2018). Topical Nanostructured Lipid Carrier Based Hydrogel of Mometasone Furoate for the Treatment of Psoriasis. Pharm. Nanotechnol..

[54] Hsueh Y.-S., Shyong Y.-J., Yu H.-C., Jheng S.-J., Lin S.-W., Wu H.-L., Tsai J.-C. (2021). Nanostructured Lipid Carrier Gel Formulation of Recombinant Human Thrombomodulin Improve Diabetic Wound Healing by Topical Administration. Pharmaceutics.

